# Depressive symptoms in Parkinson’s disease are insufficiently but more often treated than in other chronic conditions

**DOI:** 10.1038/s41531-023-00551-8

**Published:** 2023-07-14

**Authors:** Tatiana Usnich, Björn Hauptmann, Henrike Hanssen, Jannik Prasuhn, Alexander Balck, Max Borsche, Vera Tadic, Annika Klee, Greta Noblejas-Sanchez, Eva-Juliane Vollstedt, Christine Klein, Norbert Brüggemann, Meike Kasten, EPIPARK Study Group, Julia Graf, Nathalie Schell, Raluca Modreanu, Barbara Staemmler, Elena Loewin

**Affiliations:** 1 Institute of Neurogenetics, University of Lübeck, Lübeck, Germany; 2 Neurologisches Zentrum, Segeberger Kliniken Gruppe, Bad Segeberg, Schleswig-Holstein, Germany; 3 Department of Neurology, University of Lübeck, Lübeck, Germany; 4 Department of Psychiatry and Psychotherapy, University of Lübeck, Lübeck, Germany

**Keywords:** Parkinson's disease, Parkinson's disease

## Abstract

Depressive symptoms in Parkinson’s disease (PD) are multifactorial and are partly linked to the underlying dopaminergic deficit. However, at least a subset of PD patients may exhibit an unspecific depressive reaction to chronic illness. Here, we compared the prevalence and severity of depressive symptoms in PD patients and disease controls (DC). PD patients reported depressive symptoms at similar frequencies as DC but were on antidepressants, especially Mirtazapine, more frequently. Still, in both groups, a high proportion of patients with clinically significant depressive symptoms was not receiving medication. Diagnosis and treatment of depressive symptoms both in PD and DC should be improved.

Depression worsens the prognosis and quality of life of individuals with Parkinson’s disease (PD) but remains underdiagnosed and undertreated^1–4^. Depression has been reported to occur in PD more frequently than in the general population^5^. However, the prevalence of depression in patients with other chronic conditions has also been reported to be higher than in the general population^6^. To our knowledge, only a few studies compared the prevalence of depression in PD with other diseases causing motor impairment, such as arthritis, essential tremor, and dystonia, and report similar frequencies of depression in these conditions as in PD^7,8^.

Diagnosing depression in PD is challenging due to the overlap of symptoms of both conditions, fluctuating mood states and off-periods^9,10^. The pathophysiology of depression in PD is likely attributable to disturbances in dopaminergic, serotonergic, and noradrenergic pathways and is linked to the underlying biologic processes of neuronal cell loss^11^. However, some patients may exhibit more general depressive reactions due to chronic illness. Differentiation of subtypes of depressed PD patients has been proposed with a prevalence of 10.2% of PD patients suffering from depressive symptoms specifically associated with PD in contrast to 6.8% of PD patients with an unspecific depressive reaction due to a chronic progressive illness^12^.

We aimed to analyze whether depressive symptoms in PD are sufficiently recognized and treated. In addition, we investigated the prevalence and severity of depressive symptoms in PD compared to patients with motor impairment due to other chronic conditions.

## Results

### Frequency of depressive symptoms

**Fig. 1:**
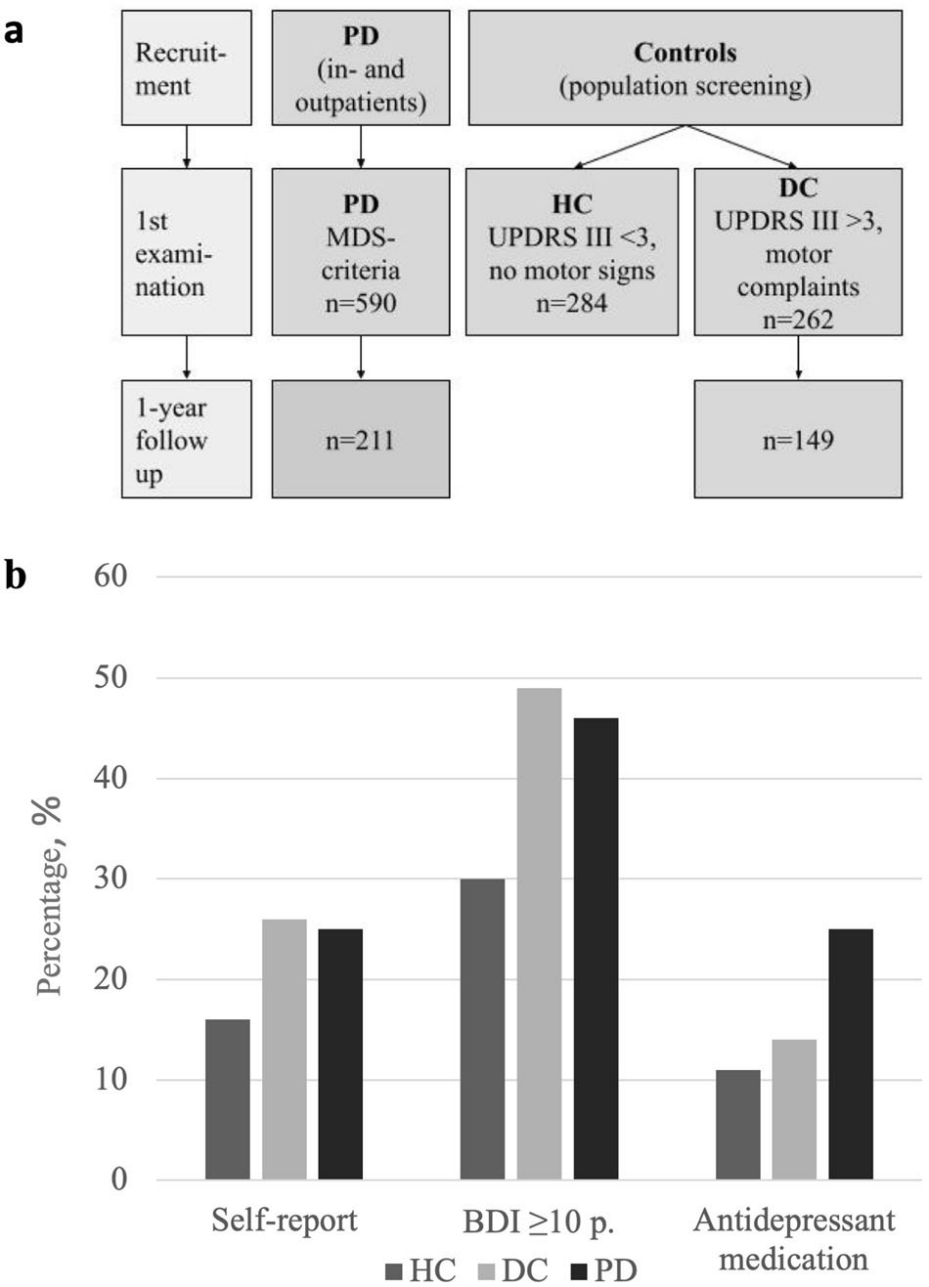
Flow chart and frequencies of depression. **a** Flow chart for the study participants. **b** Frequencies of depression based on self-report, BDI, and antidepressant use. The frequencies of depression are given in percent (%), the cut-off of the Beck Depression Inventory (BDI) 1a was >10. PD Parkinson’s disease, HC healthy controls, DC disease controls, UPDRS Unified Parkinson’s Disease Rating Scale Part III, MDS Movement Disorder Society.

PD and DC most frequently reported prior episodes of depression compared to the HC (lifetime self-report question) (Fig. 1b, Table 1). PD patients were more likely (45.8%) to have current depressive symptoms (BDI Ia≥ 10) compared to HC (29.8%), but not DC (48.6%). Stratification of BDI Ia scores into severities indicated depressive symptoms in the PD group to be predominantly mild to moderate (10–18 points) (Table 1). Severe depressive symptoms (BDI Ia≥ 18; *n* = 8 in the PD group, *n* = 13 in the DC group) were too rare for meaningful conclusions. We found no significant difference in the prevalence of depressive symptoms between PD patients receiving dopaminergic medication or not (BDI Ia ≥ 10: 173/354 (48.9%) vs. 23/43 (53.5%); *p* = 0.567).

**Table 1:** Clinical and demographic characteristics of the study participants.

Examination	HC *N* = 284	DC *N* = 262	PD *N* = 590	*P-*value
Male, *n* (%)	142 (50%)	127 (48.5%)	361 (61.3%)	**<0.001**
Age, y, mean ( ± SD)	64 ( ±7)	67 ( ±7)	67 ( ± 10)	**<0.001**
UPDRS III, mean ( ± SD)	0.9 ( ±1.1) *N* = 283	6.5 ( ±5.6)	25.6 ( ± 11.2) *N* = 580	
Hoehn and Yahr, median	-	-	2.5 *N* = 502	
Schwab and England Scale	-	-	80% (ON) *N* = 529 60% (OFF) *N* = 275	
Disease duration, y, mean ( ± SD)	-	-	7 ( ±5.8)	
LEDD, mean ( ± SD)	-	-	578 ( ±475)	
Dopamine agonists drug use, *n* (%)	-	-	317 (53.7%)	
Antidepressant drug use, *n* (%)	25/228 (11%)	33/238 (14%)	145/570 (25%)	**<0.001**
Antidepressant in therapeutic dosage, *n* (%)	10/223 (4.5%)	10/233 (4.3%)	97/570 (17%)	**<0.001**
Self-reported depression, *n* (%)	44/271 (16%)	65/245 (26%)	147/579 (25%)	**0.005**
BDI groups				
● none to minimal (0–9)	179(70.2%)	108 (51.4%)	214 (54.2%)	**<0.001**
● ≥10	76 (29.8%)	102 (48.6%)	181 (45.8%)	<**0.001**
● mild to moderate (10–18)	50 (19.6%)	66 (31.4%)	128 (32.4%)	**0.001**
● moderate to severe (19–29)	18 (7.1%)	22 (10.5%)	45 (11.4%)	0.197
● severe ( ≥30)	8 (3.1%) *N* = 255	14 (6.7%) *N* = 210	8 (2.0%) *N* = 395	**0.011**
BDI: none to minimal (0–9), *n* (%)	7/145 (4.8%)	6/97 (6.2%)	36/211 (17.1%)	**<0.001**
BDI mild to moderate symptoms (10–18), *n* (%)	7/36 (19.4%)	8/62 (12.9%)	33/121 (27.3%)	**0.063**
BDI moderate to severe symptoms (19–29), *n* (%)	5/15 (33.3%)	5/19 (26.3%)	17/44 (38.6%)	0.637
BDI severe symptoms ( ≥30), *n* (%)	5/8 (62.5%)	6/12 (50.0%)	2/7 (28.6%)	0.417
Physical health mean( ±SD), *n*	72.83 ( ± 16.32), 261	58.28 ( ± 20.46), 219	56.46 ( ± 16.89), 113	**<0.001**
Psychological mean( ±SD), *n*	71.38 ( ± 15.15), 262	64.7 ( ± 17.47), 219	65.25 ( ± 14.68), 112	**<0.001**
Social relationships mean( ±SD), *n*	66.72 ( ± 17.40), 261	62.54 ( ± 19.79), 218	65.40 ( ± 16.51), 112	**0.041**
Environment mean( ±SD), *n*	77.19 ( ± 12.72), 261	72.04 ( ± 14.37), 220	74.23 ( ± 13.51), 112	**<0.001**

*HC* Healthy controls, *DC* Disease controls, *PD* Patients with Parkinson’s disease, *n* Number, *y* Years, *SD* Standard deviation, *UPDRS* *III* Unified Parkinson’s Disease Rating Scale Part III, *LEDD* Levodopa equivalent daily dose, *BDI* Beck’s Depression Inventory, WHO QoL BREF World Health Organization Quality of Life Questionnaire.

Bold values indicate statistical significant *P* values.

### Use of antidepressants

Patients with PD were more likely to be on antidepressants (145/570 (25%)) than individuals from the other groups, with a particularly large difference in the range of mild to moderate depressive symptoms (BDI Ia = 10–18). Dosages in the therapeutic range were administered to 17% of the PD group but less than 5% of the study participants in the control groups (Table 1). In the PD group, 42/145 (29%) individuals on antidepressants received Mirtazapine (7.5 mg: *n* = 4; 15 mg: *n* = 22) and four Amitriptyline. In the DC group, only 2/33 (6%) took Mirtazapine 15 mg, and 5/33 (15%) Amitriptyline.

### Comorbidities

Patients with PD exhibited vertigo/dizziness, essential tremor, restless legs syndrome, joint diseases, back pain, thyroid disease, hyperlipidemia and cardiac arrhythmia more often than DC, whereas epilepsy, migraine, stroke, polyneuropathy, coronary heart disease and diabetes mellitus showed similar frequencies in both groups. Arterial hypertension and cancer appeared more often in the PD group compared to the DC group, however, this effect was no longer present after adjustment for age and sex (Table 2).

**Table 2:** Comorbidities of the study participants.

	DC *N* = 262	PD *N* = 590	*p*-value	*p*-value*
Neurological conditions				
Vertigo/dizziness	1 (0.4%)	55 (9.3%)	**<0.001**	**0.001**
Essential tremor	1 (0.4%)	31 (5.2%)	**<0.001**	**0.010**
Restless legs syndrome	4 (1.5%)	38 (6.4%)	**0.002**	**0.004**
Epilepsy	3 (1.1%)	8 (1.2%)	0.811	0.670
Migraine	5 (1.9%)	18 (3%)	0.351	0.210
Stroke	20 (7.6%)	44 (7.5%)	0.903	0.820
Polyneuropathy	7 (2.7%)	31 (5.2%)	0.096	0.110
Non-neurological conditions				
Joint diseases	25 (9.5%)	105 (17.8%)	**0.002**	**0.002**
Back pain	14 (5.3%)	103 (17.3%)	**<0.001**	**<0.001**
Arterial hypertension	46 (17.5%)	202 (34%)	**<0.001**	0.990
Thyroid disease	13 (5%)	85 (14.4%)	**<0.001**	**<0.001**
Hyperlipidemia	9 (3.4%)	49 (8.3%)	**0.010**	**0.014**
Cardiac arrhythmias	6 (2.3%)	49 (8.3%)	**0.001**	**0.007**
Cancer	0 (0%)	45 (7.6%)	**<0.001**	0.994
Coronary heart disease	25 (9.5 %)	62 (10.5%)	0.695	0.898
Diabetes mellitus	23 (8.8%)	65 (11%)	0.340	0.512

*DC* Disease controls, *PD* Patients with Parkinson’s disease, *p*-value* adjusted for age and sex.

Bold values indicate statistical significant *P* values.

### Quality of life

The WHO-QoL showed an equally reduced quality of life for PD and DC compared to HC. Only the physical limitations domain score was lowest in the PD group (Table 1). The strongest predictor of poor QoL in a group-spanning analysis was the presence of at least mild depressive symptoms (BDI Ia ≥ 10) (OR = 6.416; B = 1.859, 95% CI = 4.712–8.737, *p* < 0.001).

### Symptom profiles

We compared the relative contribution of individual BDI items to the total BDI score between the DC and PD groups. The PD group exhibited higher proportions of the items “tiredness” (*p* = 0.012) and “loss of weight” (*p* = 0.010) and lower contributions of the items “feelings of failure” (*p* = 0.002), “feelings of guilt” (*p* < 0.001), “self-hate” (p < 0.001), “self-accusation” (*p* = 0.008), “suicidal thoughts” (*p* = 0.021), “loss of libido” (*p* < 0.001) than the DC group.

### One-year follow-up

In total, 149 DC and 211 PD patients appeared for follow-up (mean time interval 11.9 ± 0.8 months). A similar number of persons newly developed a BDI Ia> 10 in both groups (DC: 6/75 (8.0%), *p* = 0.115; PD: 14/97 (14.4%), *p* = 0.310). Most of the PD patients on antidepressant medication during the first visit continued its use (23/34 (67.6%)), whereas 11/34 (32.4%) discontinued it. Out of 123 PD patients unmedicated during the first visit, 9 (7.3%) started antidepressant medication within the follow-up period. In the DC group, 2/7 (28.6%) on medication during the first visit discontinued the medication, and 0/31 patients who had not been on medication started it until the second visit.

## Discussion

We did not find any differences in the prevalence of depressive symptoms in PD compared to DC while confirming the higher prevalence of depressive symptoms in PD than in HC. More frequent use of antidepressants in PD may indicate partially successful treatment of depressive symptoms in PD, resulting in lower current prevalences and severities. On the other hand, PD and DC patients in our study reported similar lifetime prevalences of depression, arguing against this possible explanation. It seems more likely that neurologists address and treat depressive symptoms more often than other physicians treating DC.

Only part of the study participants received medication in the recommended therapeutic dosage. Although low dosages of antidepressants may be effective in elderly study participants, we observed low dosages more frequently in the PD group than in the DC group. Low dosages of Mirtazapine or Amitriptyline are often prescribed as a sleeping aid. Our study participants reported similar prevalences and severities of sleeping problems. Therefore, we may see an effect of low dosages of Mirtazapine prescribed as a sleeping aid in PD. Furthermore, the use of antidepressants for minimal or mild depressive symptoms is debatable as it is not supported by sufficient data.

Conflicting reports exist concerning the effect of dopaminergic medication on depressive symptoms. Previously, Pramipexole has been found to improve depressive symptoms^11,13^. However, a recent study showed no independent effect of dopaminergic medication on mood in PD when adjusted for motor improvement^14^. In our sample, we also did not find a significant difference in the prevalence of depressive symptoms between PD patients receiving dopaminergic therapy or not. Further, the quality of depressive symptoms in PD was nonspecific, with the highest contribution of “tiredness” and “loss of weight” to the total BDI in the PD group. These results may be attributable to the different subgroups of depression in PD, arguing that, at least in part, depressive symptoms in PD are not specific to the disease.

PD patients exhibited a higher burden of comorbidities than DC. Out of those, joint diseases, back pain^15^ and vertigo/dizziness^16^ have known associations with PD, and restless legs syndrome is considered a clinical manifestation in both the prodromal and the manifest phase of the disease^17^. Further, an association of PD with essential tremor has also been established^18^. In previous reports, PD patients have been found to exhibit accompanying other conditions more often than participants with other diseases presenting to primary care^19^. Similar to our results, these conditions were mostly known to be related to PD. In our analysis, after adjustment for age and sex, we found thyroid disease, hyperlipidemia and cardiac arrhythmia to be more common in the PD group than in DC. In a recent meta-analysis, thyroid disease has been found to increase the risk of PD^20^, which would explain the higher prevalence of thyroid disease in the PD group in our analysis. However, hyperlipidemia seems to be protective against developing PD^21^ and cardiac arrhythmias have rarely been reported in PD^22^. It is possible that the assessment of previous diseases is imprecise as they were self-reported by the study participants. Further, ascertainment bias is possible, as the PD group has been recruited through an outpatient and inpatient setting, but the DC group from the general population. This could partly explain the heavier burden of disease in the PD group. However, despite the higher burden of disease in PD compared to DC in our sample, PD patients showed similar prevalences of depressive symptoms as DC. This result further supports the view that depressive symptoms in PD are at least in part not specifically attributable to PD.

At a follow-up examination after one year, we observed similar proportions of patients in both groups converting to higher BDI scores and starting to take antidepressant medication. However, other study participants scored lower during the second visit and discontinued antidepressant medication, indicating a fluctuating course of depressive symptoms in PD and DC as observed in non-PD-related depression.

Mild depressive symptoms seem to be common in PD patients and are clinically relevant. This is further supported by the finding that depressive symptoms are the strongest predictor of reduced quality of life. It would thus be essential to define markers of subthreshold depression in PD and develop therapeutic recommendations.

In conclusion, DC exhibit a similar prevalence of depressive symptoms as PD patients despite the higher burden of comorbidities in PD. The prevalence of depressive symptoms in PD does not seem to be influenced by the intake of dopaminergic medication. However, PD patients use antidepressants more frequently, suggesting neurologists' awareness of depressive mood as a non-motor feature in PD. Still, many patients remain underdiagnosed and undertreated, as evidenced by a high proportion of patients with clinically significant depressive symptoms not receiving medication. Therefore, there remains room for improvement in diagnosing and treating depressive symptoms in PD and DC.

## Methods

### Study design

We analyzed data from the first two visits of a prospective, population-based cohort to study non-motor symptoms in parkinsonism (EPIPARK)^23^. We recruited non-PD participants via a mailed survey sent to 10,000 inhabitants of Lübeck, Germany, aged 50–79 years. Based on age, the residents’ registration office created a random sample from all inhabitants and provided the addresses. PD patients were recruited from the Department of Neurology at the University Medical Center Schleswig-Holstein, Campus Lübeck, Germany, and from the Neurological Center of Segeberg Clinics in Bad Segeberg, Germany (Fig. 1a).

### Ethics approval

All participants gave written informed consent after approval by the local ethics committee of the University of Lübeck (AZ22-409; AZ09-069). The study was conducted in accordance with the Declaration of Helsinki.

### Clinical examination

Neurological examinations using the Unified Parkinson’s Disease Rating Scale (UPDRS) were performed by movement disorder specialists^24^. PD was diagnosed according to the established clinical criteria for PD (United Kingdom Brain Bank Society (UKBBS) and Movement Disorder Society (MDS))^25^. Non-PD participants were classified into two groups: disease controls (DC) and healthy controls (HC) (Fig. 1a)^26–28^. The examination further included assessments of comorbidities (Table 2), medication, Levodopa equivalent daily dose^29^, and the Montreal Cognitive Assessment (MoCA). No participant in the DC group reported having dementia or other neurodegenerative conditions. We used the Beck Depression Inventory Ia (BDI Ia) to assess depressive symptoms and considered a BDI Ia score of 0–9 an indicator of none to minimal, 10–18 mild to moderate, 19–29 moderate to severe, and ≥ 30 severe depressive symptoms^30^. Participants were asked whether they had ever received a diagnosis of depression (lifetime self-report question). Subjective quality of life was assessed using the World Health Organization Quality of Life (WHO QoL) questionnaire using the 26-item version^31^. The scores of four domains (physical health, psychological, social relationships, and environment) were calculated, with higher scores corresponding to a higher quality of life. We differentiated between therapeutic and non-therapeutic doses of antidepressants according to the recommended therapeutic dosages to treat depression in adults.

### Statistical analysis

Statistical analyses using SPSS28 (IBM SPSS Statistics for Macintosh, Armonk, NY: IBM Corp) comprised chi-square tests for categorical, Mann-Whitney U tests for ordinal, and t-tests or ANOVA for continuous variables. In addition, a multivariable logistic regression model was built to define factors (age, sex, group) associated with comorbidities and quality of life. A significance threshold of *p* < 0.05 was applied.

## Data Availability

The data that support the findings of this study are available from the corresponding author upon reasonable request.
